# Investigation of the Josephin Domain Protein-Protein Interaction by Molecular Dynamics

**DOI:** 10.1371/journal.pone.0108677

**Published:** 2014-09-30

**Authors:** Marco A. Deriu, Gianvito Grasso, Ginevra Licandro, Andrea Danani, Diego Gallo, Jack A. Tuszynski, Umberto Morbiducci

**Affiliations:** 1 Institute of Computer Integrated Manufacturing for Sustainable Innovation, Department of Innovative Technologies, University of Applied Sciences and Arts of Southern Switzerland (SUPSI), Manno, Switzerland; 2 Department of Mechanical and Aerospace Engineering, Politecnico di Torino, Torino, Italy; 3 Department of Physics, University of Alberta, Edmonton, Alberta, Canada; University of Calgary, Canada

## Abstract

Spinocerebellar ataxia (SCA) 3, the most common form of SCA, is a neurodegenerative rare disease characterized by polyglutamine tract expansion and self-assembly of Ataxin3 (At3) misfolded proteins into highly organized fibrillar aggregates. The At3 N-terminal Josephin Domain (JD) has been suggested as being responsible for mediating the initial phase of the At3 double-step fibrillogenesis. Several issues concerning the residues involved in the JD’s aggregation and, more generally, the JD clumping mechanism have not been clarified yet. In this paper we present an investigation focusing on the JD protein-protein interaction by means of molecular modeling. Our results suggest possible aminoacids involved in JD contact together with local and non-local effects following JD dimerization. Surprisingly, JD conformational changes following the binding may involve ubiquitin binding sites and hairpin region even though they do not pertain to the JD interaction surfaces. Moreover, the JD binding event has been found to alter the hairpin open-like conformation toward a closed-like arrangement over the simulated timescale. Finally, our results suggest that the JD aggregation might be a multi-step process, with an initial fast JD-JD binding mainly driven by Arg101, followed by slower structural global rearrangements involving the exposure to the solvent of Leu84-Trp87, which might play a role in a second step of JD aggregation.

## Introduction

A wide range of neurodegenerative diseases is characterized by the self-assembly of specific misfolded proteins into highly organized fibrillar aggregates [1]. The most diffused family among these disorders is the polyglutamine (poly-Q) expansion disease, encompassing nine members: dentatorubral-pallidoluysian atrophy, Huntington’s disease (HD), spinal and bulbar muscular atrophy and spinocerebellar ataxia (SCA) types 1, 2, 3, 6, 7 and 17.

SCA3, also known as Machado-Joseph disease (MJD), represents the most common form of spinocerebellar ataxia. The gene associated with MJD, a pathology characterized by the poly-Q instability, is the Ataxin3 (ATXN3), located on chromosome 14 (14q32.1) [2], [3]. It encodes a 42 kDa protein (At3) consisting of the N-terminal Josephin Domain (JD), and the C-terminal unstructured tail. It is well recognised that not only a threshold of almost 51 repeats determines the pathology insurgence, but also that a correlation exists between cytosine-adenine-guanine (CAG) sequence number and both the age of onset (negative correlation) and symptoms severity (positive correlation) [4]–[8]. Many efforts have been made to better understand the mechanisms of At3 aggregation and fiber formation, because of their implication in the MJD [2], [3]. Recent findings have indicated a double-step process for At3 fibrillogenesis, composed by an initial phase JD-mediated but polyQ-independent, followed by a polyQ-dependent step [9]–[11].

The JD atomic structure has been the subject of several studies, and two different JD NMR structures (PDB entry 1YZB [12] and 2AGA [13] – UNIPROTID: P54252) are available in the literature. The two structures, both determined by NMR, differ significantly in the hairpin region (Val31-Leu62). In the 1YZB model [14], the JD is in an open semi-elongated L-shape conformation, while the 2AGA is characterized by the hairpin region packed against the rest of the structure. The dynamic behaviour of the JD has been experimentally investigated using various imaging techniques, such as fluorescence spectroscopy [15], NMR spectroscopy [16], [17], size exclusion chromatography [10], transmission electron microscopy [18] and atomic force microscopy [19].

JD is thought to have an intrinsic amyloidogenic potential [20], with an aggregation kinetics mainly driven by Ile77-Gln78, and Trp87, part of JD Ubiquitin binding site 1 (UbS1) and site 2 (UbS2), respectively [20]. Several issues concerning both residues involved in the JD’s aggregation process and, more in general, the dimerization mechanism have not been clarified yet.

In this connection, computational approaches and in particular molecular dynamics (MD) allow us to investigate protein aggregation with an atomic resolution [21], [22] and have often demonstrated to be helpful in adding valuable quantitative information to experimental data [23]–[35].

Recently, a combined experimental and computational study has provided indications on how hydrophobic/charged-surface induce structural changes of JD protein structure [23]. Specifically, residues Arg101 and Arg103 have been identified as mainly responsible for JD interaction with hydrophobic and hydrophilic surfaces [23].

In this paper we present an investigation focusing on JD-JD interactions by means of MD simulations. Our results provide an atomistic investigation of the JD-JD binding, pointing out the attention to *i*) the most likely interacting areas of the bound JD-JD complex; and *ii*) the local and non-local structural rearrangements following the JD-JD binding. We found residue Arg101 mainly involved in the JD-JD interaction surface. The JD-JD binding might also be responsible for exposure of Trp87, which has been supposed to drive JD aggregation kinetics in a recent study [20]. Moreover, our results indicate that JD binding might alter the hairpin conformation from an open-like toward a closed-like arrangement.

## Materials and Methods

The atomic structure of the JD was obtained from RCSB Protein Data Bank-PDB entry 1YZB.pdb [12]. A preliminary 100 ns MD simulation in explicitly solvated environment was carried out at 300 K in the NVT. Detailed results and comparison with experimental data are reported in a previous work [23]. Starting from the above-mentioned 100 ns equilibrated output structure, we set up two different molecular systems: 1) the single JD in explicitly modelled water [36], the so-called JD^Wat^; 2) two JDs in explicitly modelled water [36], the so-called JD-JD. The JD^Wat^ simulations have been used as comparison for conformational analysis of interacting JDs.

JD^Wat^ (system size of about 40000 interacting particles) was investigated by running 150 ns MD simulations on three “replicas” with different imposed random initial velocities by following a Maxwell-Boltzmann distribution. The last 50 ns taken from MD trajectories of these three replicas have been analyzed as ensembles for conformational analysis of the single JD in water.

JD-JD dynamics was simulated by employing the following set-up. Both JDs were randomly oriented in a box and positioned with a starting protein-protein separation distance of about ∼1 nm. Each system, fully solvated, consisted of about 50000 interacting particles. Ten replicas (Movie S1) of the JD-JD system (different initial JD orientations and atom velocities) were generated and simulated for 150 ns. The last 50 ns taken from MD trajectories of the ten replicas have been analyzed as ensemble data for analysis of interacting JDs. Alanine mutation simulations have been also carried out to verify the role of residues identified as mainly involved in JD protein-protein interaction.

All the simulations were carried out by GROMACS 4.6 package [37]. Periodic boundary conditions were applied along the xyz coordinates. The 53a6 GROMOS force field [38]–[40], was used for defining protein topology. A 500-step energy minimization by steepest descent was carried out followed by a preliminary position restrain MD of about 50 ps (with a time step of 2 fs). In details, restraining potentials were applied to the protein backbone and the MD simulation were carried out in the isothermal-isobaric (NPT) ensemble at 300 K and 1 atm. Temperature (*v-rescale*) and pressure (*berendsen*) were controlled by using weak coupling thermostats [41], [42]. Electrostatic interactions were treated by means of Particle Mesh Ewald (PME) approach. Lennard-Jones interactions were cut-off at a distance of 12 Å, with a smooth switch-off starting at a distance of 11 Å. MD were performed at 300 K in the canonical (NVT) ensemble as explained above. By associating heavy atoms and virtual site approach [38] to the LINCS constraint solver (selecting all-bonds constraints) [43], a time step of 5 fs was used. The Visual Molecular Dynamics (VMD) [44] package was employed for the visual inspection of the simulated systems. Dedicated GROMACS tools were used for a quantitative analysis in terms of Root-Mean-Square Deviation (RMSD), Root-Mean-Square Fluctuation (RMSF), Solvent Accessible Surface (SAS) [45], [46], Contact Surface, Radius of Gyration (RG) and Contact Maps. This allowed us to identify residues involved in the contact surface after the binding event. Residue mainly responsible for JD-JD interaction has been identified by contact probability plots. Contact probability for each residue was calculated using the following procedure. Trajectory snapshots are extracted in the last 50 ns of each MD simulation (e.g., for the JD-JD, one snapshot is taken every 50 ps for all the simulations in the last 50 ns). For each snapshot the distance between a residue in a monomer and all residues of the interfacing monomer is calculated. If, at least one distance value among the residue-residue distances is equal or less than a chosen threshold (0.28 nm), the residue is considered in contact with the interfacing monomer in that snapshot. The number of “contact snapshots” divided by the number of total snapshots taken out from the MD trajectories is the contact probability associated with the residue.

Analysis of secondary structure (SS) dynamics was performed by applying both the Dictionary of Protein Secondary Structure (DSSP) [47], [48], STRuctural IDEntification (STRIDE) [49], [50], and Ramachandran plots, produced by using PROCHECK [51].

## Results

### JD^wat^ simulations

The last 50 ns of the three replicas of JD^wat^ MD simulation trajectories were taken together as ensemble data for statistical analysis. Protein/Cα RMSD values ranging from 0.5 nm to 0.7 nm (Figure S1 in File S1) were detected for each simulated model, with a reasonable stability to the RMSD for the protein core. In all cases, the variation of the RMSD value for the globular core, is less then 0.1 nm in the last 50 ns, while the hairpin region (Val31-Leu62) is characterized by higher continuous RMSD fluctuations (Figure S2 in File S1). Concluding, a large contribution to the RMSD can be attributed to the hairpin region, according to the computational findings of Nicastro and coworkers [16].

**Figure 1:**
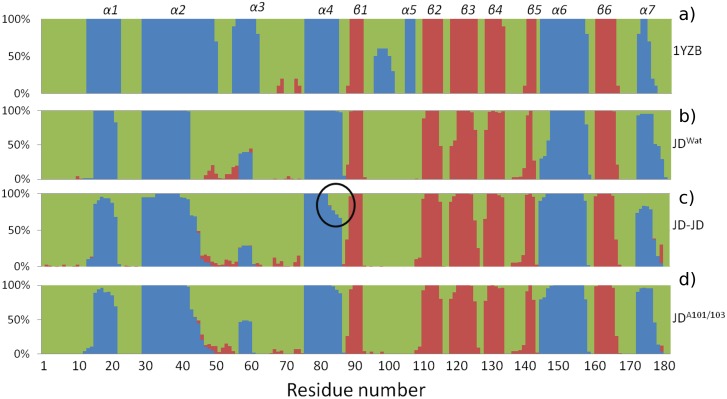
Secondary structure percentage, calculated over the available NMR configurations contained in 1YZB (a), the configurations are taken every 50 ps from JD^Wat^(b), JD-JD (c), and JD^A101/A103^ (d) in the last 50 ns of the MD trajectories. Secondary structures are indicated by different colours in the figure: green (coil), blue (helix), red (β-sheet).

Figure 1 shows the JD’s secondary structure probability, calculated by averaging the available 10 NMR configurations contained in 1YZB [12] (Figure 1a) and the configurations taken every 50 ps from JD^Wat^ trajectories (Figure 1b) between 100 and 150 ns. The JD secondary structure is highly conserved, with the exception of α5 and, partially, α3 and α6, characterized by an intrinsic tendency of helix-coil transition in the water environment.

Ramachandran plots generated for all snapshots collected indicated that more than 97% of the protein residues were always in the most-favoured and additional-allowed regions (Figure S3 in File S1) providing support for the model’s correctness.

**Figure 2:**
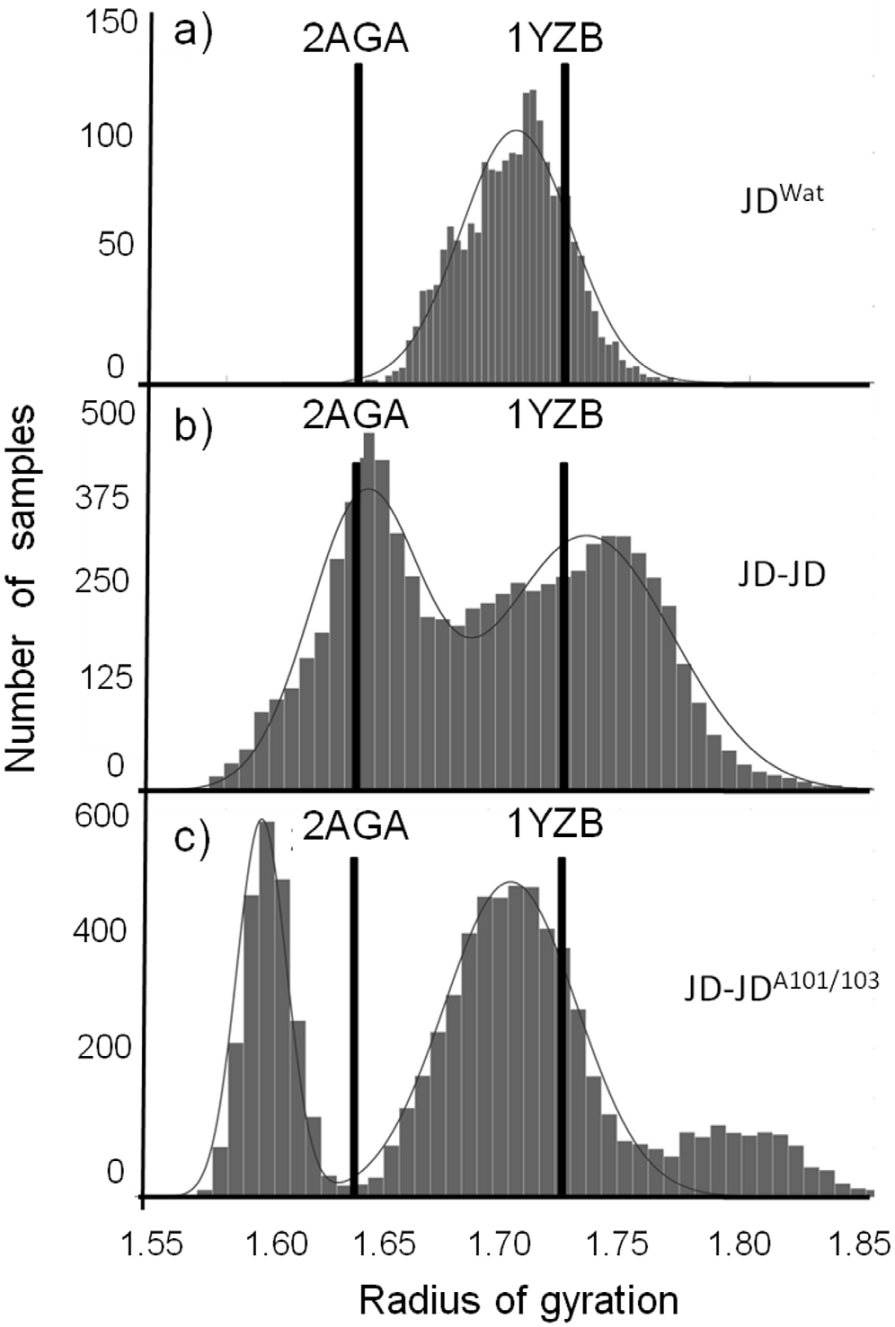
Radius of Gyration (RG) distribution of the single JD, from trajectories of JD^Wat^(a), JD-JD (b), and JD^A101/A103^ (c) simulations. The experimental RG value of 1YZB and 2AGA (open/closed hairpin, respectively) are indicated for comparison.

Figure 2a shows the Radius of Gyration (RG) probability distribution interpolated by using Gaussian interpolation. The RG distribution’s (Figure 2a), characterized by µ_RG_ = 1.71 nm and σ = 0.03 nm is in close agreement with the experimental data (PDB entry 1YZB.pdb [12], µ = 1.73 nm, σ = 0.01 nm). In Figure 2a is also indicated the RG value of the “closed-like” JD model, also solved by NMR (PDB entry 2AGA.pdb [13], µ__RG_ = 1.65 nm, σ = 0.02 nm).

### JD-JD simulations

Ten JD-JD simulations, corresponding to ten different initial JD-JD orientations, were carried out for 150 ns in the NVT ensemble. Along the overall MD simulations two distinct phases can be identified (Figure S5 in File S1). A first phase (0–100 ns), characterized by the binding event, with the two JDs approaching and non-covalently binding; a second phase (100–150 ns) with the two JDs in contact with a contact area ranging from 3 nm^2^ to 11 nm^2^, depending on the starting configuration. The last 50 ns of the ten JD-JD simulations have been considered for protein conformational analysis as a statistical data ensemble. In particular, RG and secondary structures have been calculated for each single JD (two monomers for each simulation) involved in the dimerization process. In Figure 2b the RG distributions of the JDs, interpolated by using Gaussian Interpolation, show that the mixture of two normal distributions correctly describes the sampled configurations (µ__RG1_ = 1.73 nm, σ = 0.3 nm, µ__RG2_ = 1.65 nm, σ = 0.2 nm). Interestingly, each Gaussian peak falls near the experimental RG of the two Josephin Domain NMR models (1YZB [12] and 2AGA [13]).

**Figure 3:**
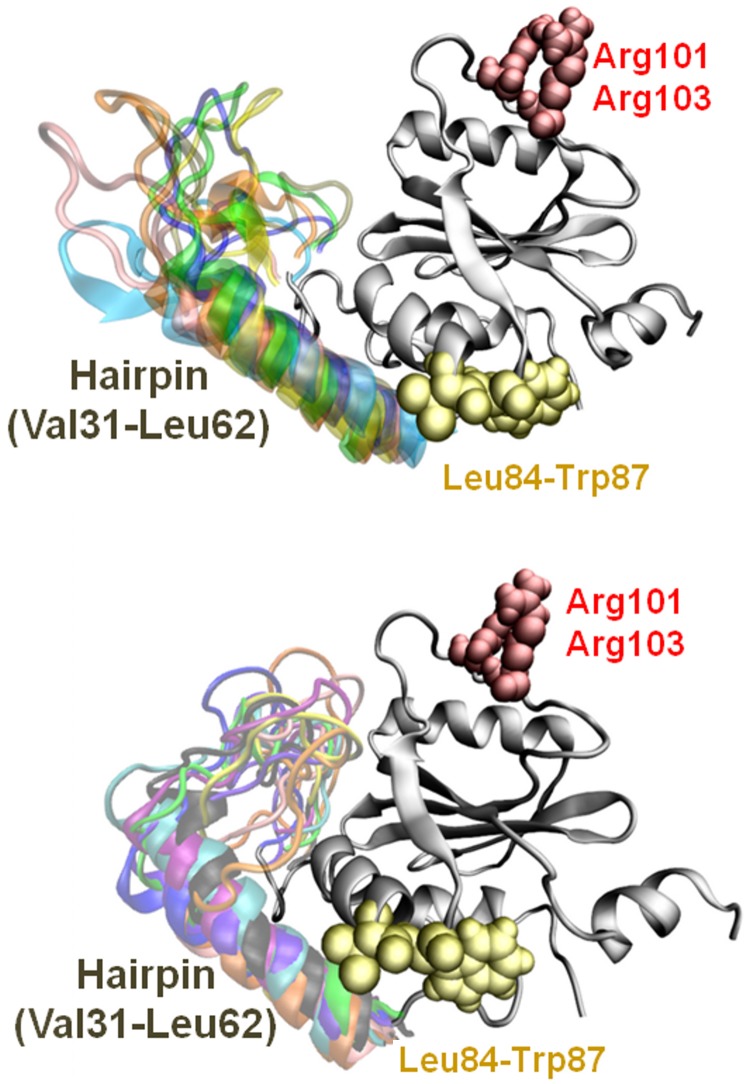
JD configuration in the open-like shape (top). The more compact (bottom) JD globular shape is the consequence of the hairpin closure dynamics. Several possible configurations of open/close hairpin are superimposed.

A visual inspection revealed the more compact globular shape (RG = 1.65 nm) as a consequence of the hairpin closure (Figure 3). At the simulated timescale, in contrast to JD^Wat^ simulations, during the dimerization process both conformations (open/closed hairpin) are sampled.

**Figure 4:**
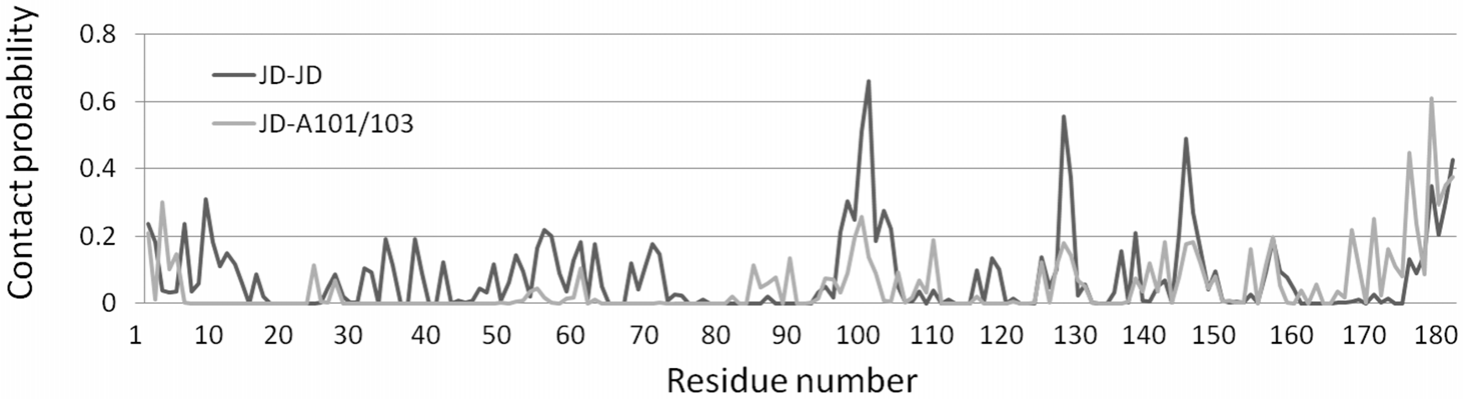
JD-JD contact probability. The inter-monomer residue-residue distance is calculated for JD-JD (black curve) and JD^A101/A103^ by applying a distance cut-off of 0.28 nm. The last 50 ns (frames taken every 50 ps) of all simulations are considered for data sampling.

We calculated the JD-JD residues’ contact probability (Figure 4) applying a distance cut-off of about 0.28 nm (roughly the diameter of a water molecule).

As a result, Arg101 has been found to be the residue most frequently involved in the JD-JD dimerization interface. In particular, Arg101 is part of the contact area over the 65% of the total sampled configurations. Two additional residues, Lys128 and Asp145, are characterized by high contact probability (55% and 50%, respectively). Moreover, also C-terminal (in particular Leu178/Arg182), seems to be involved in JD-JD interaction interface with a contact probability around 40%.

Interacting interfaces involving Arg101 are essentially characterized by charged and polar residues (Table S1 in File S1). This result may indicate a JD-JD dimerization mainly driven by electrostatic interactions, such as for example the interaction between positively charged Arg101 of one monomer and negative Asp57 of the second one.

**Figure 5:**
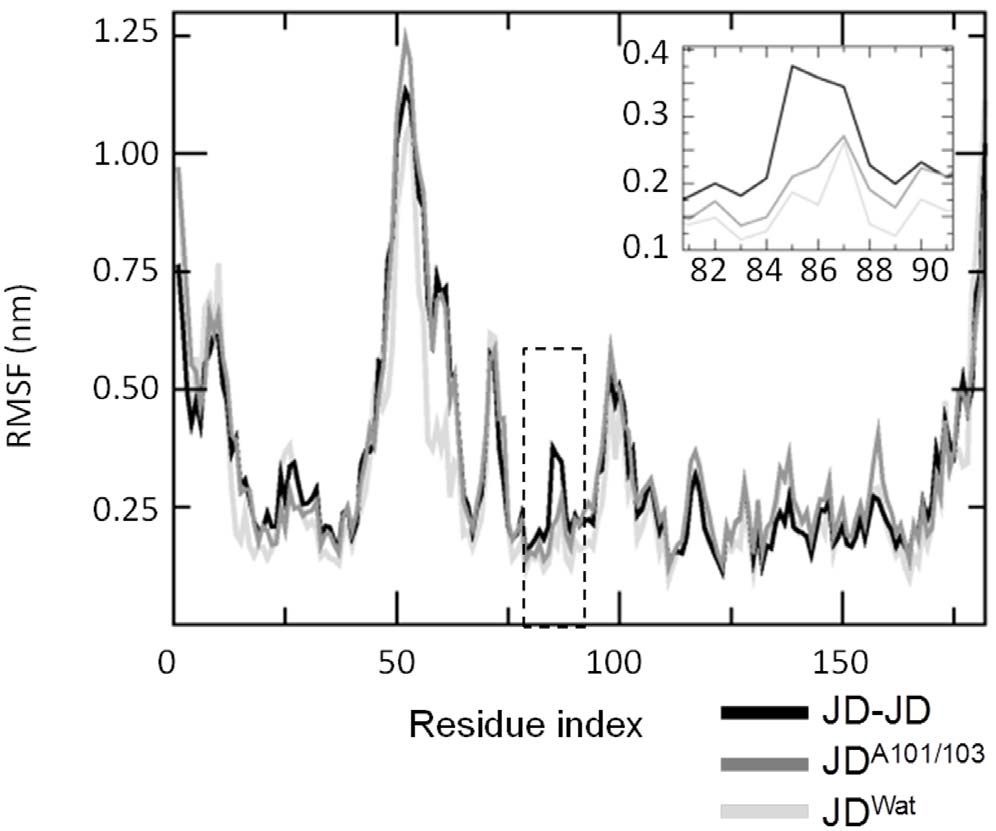
Single JD root-mean-square fluctuations (RMSF). Fluctuations of α4 are zoomed in the top-right panel where it is worth noticing that residues Leu84-Trp87 increase the RMSF as a consequence of JD dimerization (black curve). After Ala-mutation (gray curve) on Arg101-/Arg103 the RMSF value decrease to the value achieved by JD^Wat^ (light gray curve).

**Figure 6:**
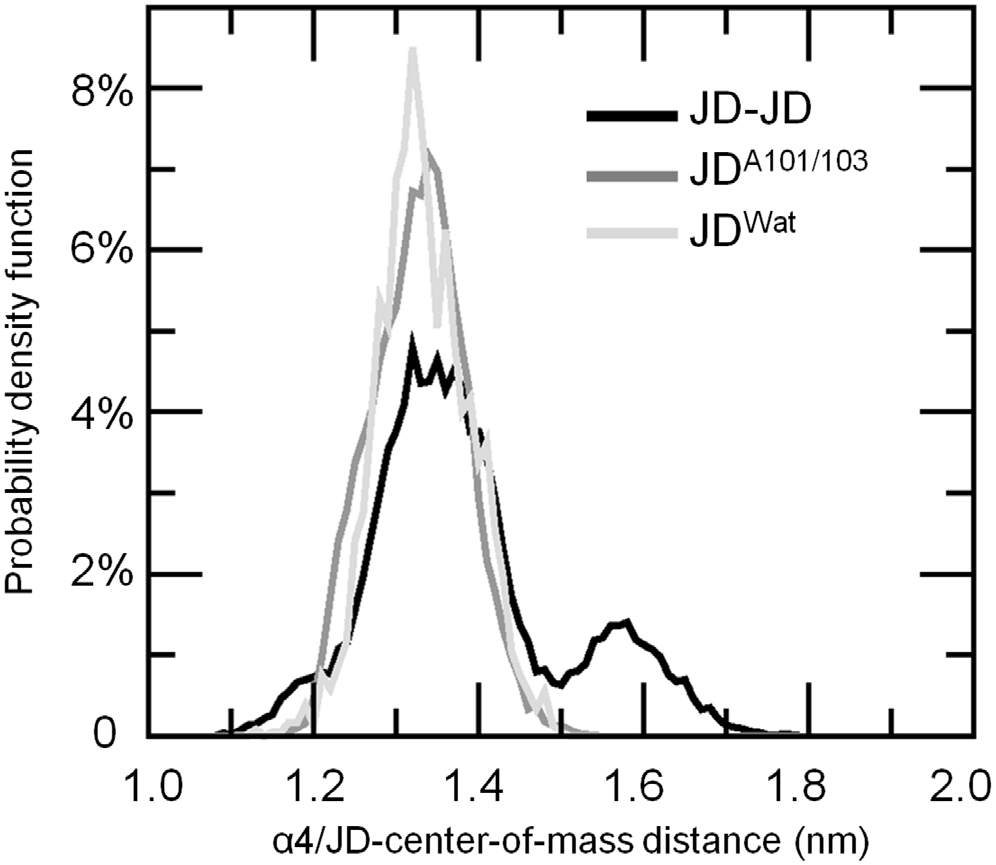
α4-Protein/centre of mass (COM) distribution. Similar behavior (Gaussian-like) is shown for JD^Wat^ (light grey curve) and JD^A101/A103^ (gray curve). A subset of sampled JD-JD configurations (black curve) reveals α4 moving far from the protein centre of mass.

**Figure 7:**
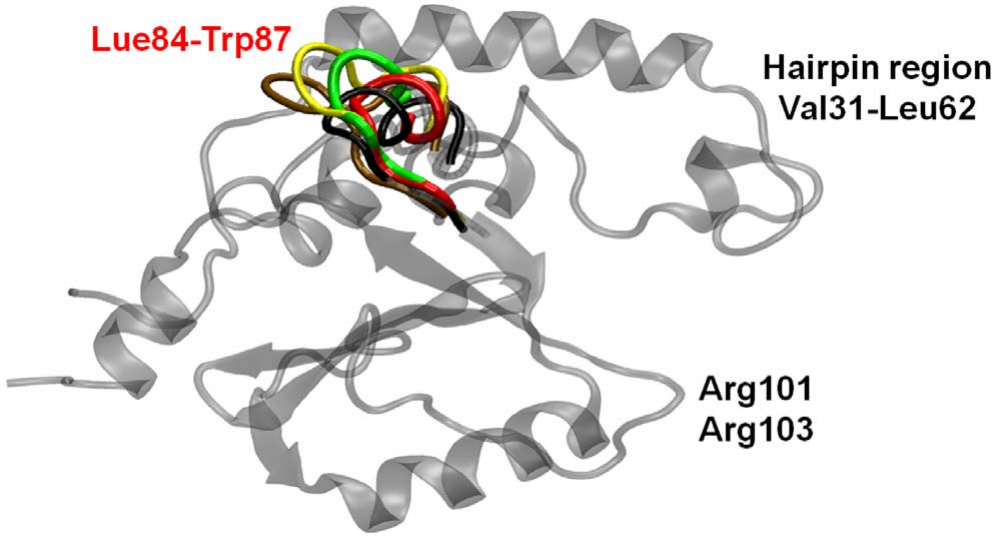
The visual inspection of MD trajectories reveals Leu84-Trp87 conformational change and solvent exposure after the JD-JD binding. Four snapshots throughout the MD are shown (red, green, yellow, and brown); the 1YZB conformation of Leu84-Trp87 is shown in black. The JD structure is transparent and grey. Hairpin region and Arg101/Arg103 residues are labeled.

Secondary structure analysis (Figure 1c) highlights a remarkable (35% of the sampled JD configurations) helix-coil transition involving α4, and in particular Leu84-Trp87. The structural flexibility characterizing Leu84-Trp87 during the aggregation process, caused by the partial unfolding of the site, is also confirmed by a significant raise in RMSF if compared with JD^Wat^ simulations (RMSF α4^JWat^ = 0.2 nm, RMSF α4 = 0.38 nm), as shown in Figure 5. The RMSF increase of α4 is directly related to a higher solvent exposure after a partial unfolding. The α4 (in particular Leu84-Trp87) exposure is highlighted by plotting the α4/protein-centre-of-mass distance distribution, compared with JD^Wat^ data (Figure 6). It is worth noticing that a subset of sampled configurations reveal α4 moving far from the protein centre of mass (Figure 7 and Movie S2), and more exposed to the solvent.

### JD^A101/A103^ simulations

Results from wild JD-JD simulation have evidenced a leading role of Arg101 in the JD dimerization mechanism. This result is in close agreement with a very recent experimental/computational study [23] demonstrating Arg101/Arg103 as mainly responsible for JD interaction with hydrophobic/hydrophilic substrates.

To carefully test this hypothesis, we run again the same set of ten JD-JD simulations (150 ns, starting from exactly the same initial conditions) by replacing the native Arg101/Arg103 residues with Alanine (we will refer to this set of simulations using the designation JD^A101/A103^).

Comparison with JD-JD simulations, in terms of the RG distribution (Figure 2b and c), provides evidence that Arg101/Arg103 mutations have a global significant influence on the JD structural properties, while secondary structure is still conserved (Figure 1d). In case of Alanine-mutation, Gaussian peaks (µ__RG1_ = 1.71 nm, σ = 0.2 nm, µ__RG2_ = 1.61 nm, σ = 0.1 nm) no longer match the experimental data. The mutations have a strong effect on the JD-JD interaction propensity (Figure 4), providing a generalized reduction of the contact probability distribution, not only on the mutated residues Ala101 (from 65% to 25%), but also on residues Lys128 (from 55% to 18%) and Asp145 (from 50% to 18%). The dimerization interface contains mainly the unstructured coil (residues Leu178-Arg182) with a contact probability value of 60% over the sampled configurations. It worth mentioning that 50% of interacting residues are from the C-terminus of the interfacing monomer. Hence, a mutated JD-JD interface is principally characterized by tail-tail interactions (Table S2 in File S1).

Moreover, our data show that Arg101/Arg103 substitution did not result in any α4 unfolding tendency (Figure 1) as shown for the JD-JD simulations. This finding is also consistent with the RMSF curve (Figure 5) highlighting a remarkable reduction of α4 fluctuations in case of mutated JD (RMSF α4^JD-JD^ = 0.38 nm, RMSF α4^JD-A103/103^ = 0.2 nm) and α4 exposure (Figure 6) with respect to the JD-JD simulations. In particular, the α4/protein-centre-of-mass distance distribution is characterized by the same curve shape and value as JD^Wat^ sampled configurations.

## Discussion

In this study, molecular dynamics has been used to investigate JD protein-protein interactions. Our simulations have been based on the 1YZB model rather than 2AGA. The choice of the 1YZB model rather than 2AGA has been dictated by a previously published work of Nicastro et al. [14], providing a validation of the 1YZB model through a quality, accuracy and mutual consistency analysis of the Josephin Domain available structures (1YZB and 2AGA).

We carried out 150 ns MD simulations of 2 JDs in the water environment, with 10 different initial JD-JD orientations, for a total simulation time of 1.5 µs. While atomistic models describing the JD-JD complex are not available in literature, a clear identification of the most likely JD interacting sites is here provided. JD-JD models are also provided as supporting information (File S2). Moreover, the last 50 ns of MD trajectories for each JD were analyzed as ensemble, allowing the description of the protein conformational changes characterizing the dimerization process. Our results suggest Arg101, Lys128 and Asp145 as the most likely JD-JD interacting sites. In particular, our findings show that Arg101 plays a major role in the JD-JD interacting interfaces, given that it is involved in the 65% of the sampled JD-JD binding surface. A very recent computational and experimental study [23] has highlighted the tendency of the JD to interact with gold and mica surfaces through Arg101/Arg103 residues. These results suggest a key role for this site in approaching and binding other proteins or interacting surfaces [23]. In order to further confirm our assumption, we considered a JD^A101/A103^ mutant, in which the native residues are replaced by Alanine. Starting from the same 10 JD-JD initial orientation (and same starting conditions), the JD^A101/A103^ dimerization was again investigated through 150 ns MD simulations, for a total simulation time of 1.5 µs. It was observed that Arginine substitution dramatically reduces the JD-JD contact probability not only on the mutated residues, but also on Lys128 and Asp145 (Figure 4). It is opinion of the authors that this evidence strongly confirms the critical role of the Arg101/Arg103 in enhancing the JD interaction propensity. Instead, mutated intermonomer interface is principally characterized by C-terminal interactions.

A recent experimental study [17], aimed at investigating the JD’s aggregation-prone regions, has proposed the sites Ile77/Gln78 and Trp87, part of the Ubiquitin binding site 1 (UbS1) and site 2 (UbS2) as having the highest aggregation propensity. In addition, Masino and co-workers [14] have provided evidence that both aberrant aggregation and physiological function involve the same sequence patterns. Hence, the protein’s non-pathological deubiquitinating function [13], [17] can play an active role in preventing aberrant fibrillization.

On the basis of our data, we did not identify Ile77/Gln78 and Trp87 residues as part of the JD-JD contact surface (Figure 4). However, α4 (and in particular Leu84-Trp87) has shown the propensity to undergo high conformational changes as a consequence of the JD-JD binding in a subset of sampled monomer conformations in JD-JD simulations. The secondary structure analysis has highlighted a remarkable helix-coil transition involving Leu84-Trp87 (Figure 1). Moreover, conformational changes with consequent exposure of the site was detected (Figure 5, Figure 6). The α4 helical loss and its solvent exposure, was detected only after the binding event. The α4 shift is probably an indirect effect of the dimerization depending on many variables. In particular, the JD binding interface and direct effects on motion of residues pertaining to JD-JD contact interfaces might finally affect the motion of α4.

Our data highlighted that Arg101 plays a dominant role in the JD-JD contact surface (Figure 4), whereas Arg101/Arg103 mutations significantly influence the contact probability values, not only on the mutated residues Ala101/Ala103 (from 65% to 25%), but also on residues Lys128 (from 55% to 18%) and Asp145 (from 50% to 18%). On the other hand, Arg101 *per se*, has shown to not be directly responsible for affecting the α4 conformational changes, as demonstrated by the comparison between JD^Wat^ and JD-JD simulations (Figure 1, Figure 5, Figure 6). Hence, Arg101 may have an indirect role in the α4 conformational changes. This is confirmed by the fact that all JD dimers characterized by α4 exposure are also characterized by Arg101 involved in the binding interface.

Our work suggests the JD aggregation might be a multi-step process with an initial fast electrostatic JD-JD binding, mainly driven by Arg101, followed by slower structural global rearrangements. In this process, α4 (and in particular Leu84-Trp87) might play a role in a second step of aggregation, being totally exposed to the solvent only after the JD-JD binding. Further investigations are needed to verify this hypothesis.

Our results also suggest the JD binding playing a role in altering JD monomer conformation. The JD solution structure is still a debated topic, especially as regards the hairpin region [14], since two different NMR structures are available in the literature [12], [13]. In general agreement, the two structures differ significantly in the hairpin region (Val31-Leu62): in the open-like 1YZB the hairpin is flexible and protruding out in solution, thus creating a cleft in which other ligands could be inserted, whereas in the closed-like 2AGA, the hairpin is close to the globular domain. Our data (Figure 2a), have shown, that JD-JD binding may destabilize the JD monomer structure, therefore shifting a open-like hairpin (1YZB) to a closed-like hairpin structure (2AGA).

Data coming from our JD^Wat^ simulations, have shown sampled conformation only close to the 1YZB. However, considering that the sampling of JD^Wat^ is based on 3 simulation of 150 ns duration and that the starting configuration was 1YZB, this result does not allow us to conclude that the 1YZB state has a lower free energy than the 2AGA. To prove the 1YZB as more likely accessible by classical MD, it would be necessary to show not only that there is more sampling in the 1YZB state than in the 2AGA state, but also that many transitions between the states represented by 1YZB and 2AGA are sampled during the simulation. In a longer time of observation, the JD alone in water environment (JD^wat^) might undergo structural rearrangements providing a deeper sampling of the state space, including the closed-like structure. For this reason, data obtained from JD^Wat^ simulations should be more suitable as a comparison term for the JD-JD simulations. Nevertheless, our data highlighted that the closed like configuration is sampled in the case of JD-JD simulations. Considering that JD^Wat^ and JD-JD simulations have the same monomer starting configuration (1YZB), our results suggest that the dimerization event might drive the opening/closing kinetics of the hairpin, destabilizing the JD structure during or after the binding. The closed-like structure, achieved after the binding event (Figure 2b), is in agreement with the NMR model provided by Mao and coworkers [13]. Outcomes of this work highlight the strong reduction in sampling ability of the closed-like conformation (which generally follows the JD binding) and also provide new information based on the investigation of the hairpin region’s dynamics on a timescale of hundreds of nanoseconds.

The intrinsic flexibility of the hairpin in the free JD, might be relevant to optimize the relative JD-Ub orientation through an excellent sampling of the space [12], [14], [16]. Moreover, JD hairpin plays an important physiological role in target recognition defining the specificity of the Ub-hydrolase activity. Further investigations are needed, especially involving longer time scales and more robust sampling methods to better elucidate the hairpin dynamics behavior, and its local and non-local motion correlation to other parts of the monomer.

In particular, the haipin closure dynamics might be related to the dynamics of region Asp57-Leu62 (α3, Figure 1). In both JD^Wat^ and JD-JD (wild/mutated) dynamics, the region α3, seems characterized by an intrinsic tendency of helix-coil transitions in the water environment. It can be noticed that, the lack of the helical region α3 highlighted in Figure 1, involves 50% of the sampled configurations. In other words, a metastable secondary structure characterizes the helix α3 in all cases. However, Figure 5 shows that the same region has RMSF values that are very different in a single JD structure or in an aggregated JD pair (wild/mutated JD). The reason for the different RMSF values between the single and the aggregated JD might lie in the closure dynamics of the hairpin. In particular, the protein structural ensemble of the JD-JD and JD^A101/A103^ includes JD configurations characterized by the hairpin in open-like, closed-like and intermediate states (Figure 7). Only the intermediate state is responsible for higher fluctuation values, with respect to the open-like and the closed-like conformation (Figure S8 in File S1), thus suggesting a relationship between hairpin closure and α3 fluctuations.

## Conclusion

In the present study the JD protein-protein interaction has been investigated by molecular dynamics. Our data have highlighted residues Arg101, Lys128 and Asp145 as mainly involved in the JD-JD contact interface. JD dimer models are provided as supporting information to this paper. Moreover, our results highlighted that, as a consequence of the JD-JD binding, α4 (and in particular Leu84-Trp87) may undergo conformational changes followed by a total exposure to the solvent. Based on the results obtained in this study, we propose that, although the peptide sequence Leu84-Trp87 has been indicated as relevant for JD aggregation in recent publications on this topic, it might play a role in a second step of the JD aggregation, the first step of the JD-JD binding being mainly mediated by other residues such as Arg101. Further work is required to confirm our hypothesis. Moreover it is necessary to better clarify the dynamic behavior of Leu84-Trp87 region, whose key aggregation role was suggested by previous publications in this area [17]. Moreover, *ad hoc* experimental studies aimed to validate the direct involvement of Arg101 in the JDs binding mechanism are needed.

In conclusion, our computational results could also be a starting point for a better understanding of SCA3 molecular pathogenesis, through a deeper investigation of the JD role into the At3 aggregation by considering also the presence of polyQ expanded tracts.

## Supporting Information

File S1 **Figures, tables and additional information to support “MATERIALS AND METHODS” and “RESULTS” sections of this paper.**
(PDF)

File S2 **Ten docked JD-JD models (Protein Data Bank format).** The last frame of each JDJD simulation is provided together with an estimation of the associated binding free energy.
(PDB)

Movie S1 **The movie shows the ten starting configurations for the JD-JD systems.** Each configuration is shown as a trajectory step.
(MP4)

Movie S2 **The movie shows α4 moving apart from the protein centre of mass as a consequence of the JD-JD binding.**
(MP4)

## References

[1] Chiti F, Dobson CMC (2006) Protein misfolding, functional amyloid, and human disease. Annu Rev Biochem 75: 333–366 Available: http://www.annualreviews.org/doi/pdf/10.1146/annurev.biochem.75.101304.123901 Accessed 4 December 2012. 16756495 10.1146/annurev.biochem.75.101304.123901

[2] Kawaguchi Y, Okamoto T, Taniwaki M, Aizawa M, Inoue M, et al (1994) CAG expansions in a novel gene for Machado-Joseph disease at chromosome 14q32.1. Nat Genet 8: 221–228 Available: http://www.tmd.ac.jp/med/phy2/Rindokupapers/CAG.pdf Accessed 10 December 2012. 7874163 10.1038/ng1194-221

[3] Takiyama Y, Nishizawa M, Tanaka H, Kawashima S, Sakamoto H, et al (1993) The gene for Machado–Joseph disease maps to human chromosome 14q. Nat Genet 4: 300–304 Available: http://www.nature.com/ng/journal/v4/n3/abs/ng0793-300.html Accessed 10 December 2012. 8358439 10.1038/ng0793-300

[4] Dürr A, Stevanin G, Cancel G, Duyckaerts C, Abbas N, et al (1996) Spinocerebellar ataxia 3 and Machado-Joseph disease: clinical, molecular, and neuropathological features. Ann Neurol 39: 490–499 Available: http://www.ncbi.nlm.nih.gov/pubmed/8619527 Accessed 9 December 2013. 8619527 10.1002/ana.410390411

[5] Ranum LP, Lundgren JK, Schut LJ, Ahrens MJ, Perlman S, et al (1995) Spinocerebellar ataxia type 1 and Machado-Joseph disease: incidence of CAG expansions among adult-onset ataxia patients from 311 families with dominant, recessive, or sporadic ataxia. Am J Hum Genet 57: 603–608 Available: http://www.pubmedcentral.nih.gov/articlerender.fcgi?artid=1801263&tool=pmcentrez&rendertype=abstract Accessed 9 December 2013. 7668288 PMC1801263

[6] Zoghbi HY, Orr HT (2000) Glutamine repeats and neurodegeneration. Annu Rev Neurosci 23: 217–247 Available: http://www.ncbi.nlm.nih.gov/pubmed/10845064 Accessed 9 December 2013. 10845064 10.1146/annurev.neuro.23.1.217

[7] Maciel P, Gaspar C, DeStefano AL, Silveira I, Coutinho P, et al (1995) Correlation between CAG repeat length and clinical features in Machado-Joseph disease. Am J Hum Genet 57: 54–61 Available: http://www.pubmedcentral.nih.gov/articlerender.fcgi?artid=1801255&tool=pmcentrez&rendertype=abstract Accessed 12 December 2013. 7611296 PMC1801255

[8] Riess O, Rüb U, Pastore A, Bauer P, Schöls L (2008) SCA3: neurological features, pathogenesis and animal models. Cerebellum 7: 125–137 Available: http://www.ncbi.nlm.nih.gov/pubmed/18418689 Accessed 12 December 2013. 18418689 10.1007/s12311-008-0013-4

[9] Ellisdon AM, Pearce MC, Bottomley SP (2007) Mechanisms of ataxin-3 misfolding and fibril formation: kinetic analysis of a disease-associated polyglutamine protein. J Mol Biol 368: 595–605 Available: http://www.sciencedirect.com/science/article/pii/S0022283607002392 Accessed 28 May 2014. 17362987 10.1016/j.jmb.2007.02.058

[10] Ellisdon AM, Thomas B, Bottomley SP (2006) The two-stage pathway of ataxin-3 fibrillogenesis involves a polyglutamine-independent step. J Biol Chem 281: 16888–16896 Available: http://www.jbc.org/content/281/25/16888.short Accessed 28 May 2014. 16624810 10.1074/jbc.M601470200

[11] Saunders HM, Gilis D, Rooman M, Dehouck Y, Robertson AL, et al (2011) Flanking domain stability modulates the aggregation kinetics of a polyglutamine disease protein. Protein Sci 20: 1675–1681 Available: http://onlinelibrary.wiley.com/doi/10.1002/pro.698/full Accessed 28 May 2014. 21780213 10.1002/pro.698 PMC3218360

[12] Nicastro G, Menon RP, Masino L, Knowles PP, McDonald NQ, et al (2005) The solution structure of the Josephin domain of ataxin-3: structural determinants for molecular recognition. Proc Natl Acad Sci U S A 102: 10493–10498 Available: http://www.pnas.org/cgi/content/abstract/102/30/10493 Accessed 20 October 2011. 16020535 10.1073/pnas.0501732102 PMC1180765

[13] Mao Y, Senic-Matuglia F, Di Fiore PP, Polo S, Hodsdon ME, et al (2005) Deubiquitinating function of ataxin-3: insights from the solution structure of the Josephin domain. Proc Natl Acad Sci U S A 102: 12700–12705 Available: http://www.pubmedcentral.nih.gov/articlerender.fcgi?artid=1188261&tool=pmcentrez&rendertype=abstract Accessed 10 March 2014. 16118278 10.1073/pnas.0506344102 PMC1188261

[14] Nicastro G, Habeck M, Masino L, Svergun DI, Pastore A (2006) Structure validation of the Josephin domain of ataxin-3: conclusive evidence for an open conformation. J Biomol NMR 36: 267–277 Available: http://www.ncbi.nlm.nih.gov/pubmed/17096206 Accessed 10 March 2014. 17096206 10.1007/s10858-006-9092-z

[15] Marchal S, Shehi E, Harricane M-CM, Fusi P, Heitz F, et al (2003) Structural instability and fibrillar aggregation of non-expanded human ataxin-3 revealed under high pressure and temperature. J Biol Chem 278: 31554–31563 Available: http://www.jbc.org/content/278/34/31554.short Accessed 4 December 2012. 12766160 10.1074/jbc.M304205200

[16] Nicastro G, Masino L, Esposito V, Menon RP, De Simone A, et al (2009) Josephin domain of ataxin-3 contains two distinct ubiquitin-binding sites. Biopolymers 91: 1203–1214 Available: http://www.ncbi.nlm.nih.gov/pubmed/19382171 Accessed 8 November 2012. 19382171 10.1002/bip.21210

[17] Laura M, Giuseppe N, Lesley C, Michele V, Annalisa P, et al (2011) Functional interactions as a survival strategy against abnormal aggregation. FASEB J 25: 45–54 Available: http://www.fasebj.org/content/25/1/45.short Accessed 4 December 2012. 20810784 10.1096/fj.10-161208 PMC3005437

[18] Chow MKM, Paulson HL, Bottomley SP (2004) Destabilization of a Non-pathological Variant of Ataxin-3 Results in Fibrillogenesis via a Partially Folded Intermediate: A Model for Misfolding in Polyglutamine Disease. J Mol Biol 335: 333–341 Available: http://www.sciencedirect.com/science/article/pii/S0022283603011811 Accessed 28 May 2014. 14659761 10.1016/j.jmb.2003.08.064

[19] Natalello A, Frana AMA, Relini A, Apicella A, Invernizzi G, et al (2011) A major role for side-chain polyglutamine hydrogen bonding in irreversible ataxin-3 aggregation. PLoS One 6: 10 Available: http://dx.plos.org/10.1371/journal.pone.0018789 Accessed 4 December 2012. 10.1371/journal.pone.0018789 PMC3076451 21533208

[20] Masino L, Nicastro G, Menon RP, Dal Piaz F, Calder L, et al (2004) Characterization of the structure and the amyloidogenic properties of the Josephin domain of the polyglutamine-containing protein ataxin-3. J Mol Biol 344: 1021–1035 Available: http://www.sciencedirect.com/science/article/pii/S0022283604012239 Accessed 28 May 2014. 15544810 10.1016/j.jmb.2004.09.065

[21] Gsponer J, Haberthür U, Caflisch A (2003) The role of side-chain interactions in the early steps of aggregation: Molecular dynamics simulations of an amyloid-forming peptide from the yeast prion Sup35. Proc Natl Acad Sci U S A 100: 5154–5159 Available: http://www.pubmedcentral.nih.gov/articlerender.fcgi?artid=154314&tool=pmcentrez&rendertype=abstract Accessed 27 November 2013. 12700355 10.1073/pnas.0835307100 PMC154314

[22] De Simone A, Kitchen C, Kwan AH, Sunde M, Dobson CM, et al (2012) Intrinsic disorder modulates protein self-assembly and aggregation. Proc Natl Acad Sci U S A 109: 6951–6956 Available: http://www.pubmedcentral.nih.gov/articlerender.fcgi?artid=3344965&tool=pmcentrez&rendertype=abstract Accessed 11 November 2013. 22509003 10.1073/pnas.1118048109 PMC3344965

[23] Apicella A, Soncini M, Deriu MA, Natalello A, Bonanomi M, et al (2013) A hydrophobic gold surface triggers misfolding and aggregation of the amyloidogenic Josephin domain in monomeric form, while leaving the oligomers unaffected. PLoS One 8: e58794 Available: http://dx.plos.org/10.1371/journal.pone.0058794 Accessed 25 March 2013. 23527026 10.1371/journal.pone.0058794 PMC3602447

[24] Nury H, Poitevin F, Van Renterghem C, Changeux J-P, Corringer P-J, et al (2010) One-microsecond molecular dynamics simulation of channel gating in a nicotinic receptor homologue. Proc Natl Acad Sci U S A 107: 6275–6280 Available: http://www.pubmedcentral.nih.gov/articlerender.fcgi?artid=2852019&tool=pmcentrez&rendertype=abstract Accessed 13 December 2013. 20308576 10.1073/pnas.1001832107 PMC2852019

[25] Sugita Y, Ikeguchi M, Toyoshima C (2010) Relationship between Ca2+-affinity and shielding of bulk water in the Ca2+-pump from molecular dynamics simulations. Proc Natl Acad Sci U S A 107: 21465–21469 Available: http://www.pubmedcentral.nih.gov/articlerender.fcgi?artid=3003049&tool=pmcentrez&rendertype=abstract Accessed 13 December 2013. 21098671 10.1073/pnas.1015819107 PMC3003049

[26] Hsin J, Gopinathan A, Huang KC (2012) Nucleotide-dependent conformations of FtsZ dimers and force generation observed through molecular dynamics simulations. Proc Natl Acad Sci U S A 109: 9432–9437 Available: http://www.pubmedcentral.nih.gov/articlerender.fcgi?artid=3386107&tool=pmcentrez&rendertype=abstract Accessed 13 December 2013. 22647609 10.1073/pnas.1120761109 PMC3386107

[27] Hornak V, Okur A, Rizzo RC, Simmerling C (2006) HIV-1 protease flaps spontaneously open and reclose in molecular dynamics simulations. Proc Natl Acad Sci U S A 103: 915–920 Available: http://www.pubmedcentral.nih.gov/articlerender.fcgi?artid=1347991&tool=pmcentrez&rendertype=abstract Accessed 12 December 2013. 16418268 10.1073/pnas.0508452103 PMC1347991

[28] Invernizzi G, Lambrughi M, Regonesi ME, Tortora P, Papaleo E (2013) The conformational ensemble of the disordered and aggregation-protective 182–291 region of ataxin-3. Biochim Biophys Acta 1830: 5236–5247 Available: http://www.ncbi.nlm.nih.gov/pubmed/23891935 Accessed 26 February 2014. 23891935 10.1016/j.bbagen.2013.07.007

[29] Deriu MA, Shkurti A, Paciello G, Bidone TC, Morbiducci U, et al (2012) Multiscale modeling of cellular actin filaments: from atomistic molecular to coarse-grained dynamics. Proteins 80: 1598–1609 Available: http://onlinelibrary.wiley.com/doi/10.1002/prot.24053/full Accessed 26 March 2013. 22411308 10.1002/prot.24053

[30] Deriu MA, Bidone TC, Mastrangelo F, Di Benedetto G, Soncini M, et al (2011) Biomechanics of actin filaments: a computational multi-level study. J Biomech 44: 630–636 Available: http://www.ncbi.nlm.nih.gov/pubmed/21130998 Accessed 12 April 2013. 21130998 10.1016/j.jbiomech.2010.11.014

[31] Enemark S, Deriu MA, Soncini M, Redaelli A (2008) Mechanical model of the tubulin dimer based on molecular dynamics simulations. J Biomech Eng 130: 041008 Available: http://www.ncbi.nlm.nih.gov/pubmed/18601450 Accessed 29 November 2010. 18601450 10.1115/1.2913330

[32] Deriu MA, Soncini M, Orsi M, Patel M, Essex JW, et al (2010) Anisotropic elastic network modeling of entire microtubules. Biophys J 99: 2190–2199 Available: http://www.pubmedcentral.nih.gov/articlerender.fcgi?artid=3042574&tool=pmcentrez&rendertype=abstract Accessed 4 April 2011. 20923653 10.1016/j.bpj.2010.06.070 PMC3042574

[33] Soncini M, Votta E, Aprodu I, Enemark S, Redaelli ACL, et al (2009) Microtubule-Kinesin Mechanics by Molecular Modeling. Biophys Rev Lett 04: 45–61 Available: http://www.worldscinet.com/brl/04/0401n02/S1793048009000922.html Accessed 11 December 2013.

[34] Soncini M, Vesentini S, Ruffoni D, Orsi M, Deriu MA, et al (2007) Mechanical response and conformational changes of alpha-actinin domains during unfolding: a molecular dynamics study. Biomech Model Mechanobiol 6: 399–407 Available: http://www.springerlink.com/index/p732734133471443.pdf Accessed 21 June 2011. 17115122 10.1007/s10237-006-0060-z

[35] Deriu MA, Enemark S, Soncini M, Montevecchi FM, Redaelli A (2007) Tubulin: from atomistic structure to supramolecular mechanical properties. J Mater Sci 42: 8864–8872 Available: http://www.springerlink.com/index/a3340u7j202l3641.pdf Accessed 26 March 2013.

[36] Berendsen HJC, Postma JPM, Van Gunsteren WF, Hermans J (1981) Interaction models for water in relation to protein hydration. In: Pullman B, editor. Intermolecular Forces. Reidel, Vol. 11: 331–338 doi:10.1111/j.1574-695X.1996.tb00128.x

[37] Hess B, Kutzner C, van der Spoel D, Lindahl E (2008) GROMACS 4: Algorithms for Highly Efficient, Load-Balanced, and Scalable Molecular Simulation. J Chem Theory Comput 4: 435–447 Available: http://pubs.acs.org/doi/abs/10.1021/ct700301q. 26620784 10.1021/ct700301q

[38] Van Der Spoel D, Lindahl E, Hess B, Groenhof G, Mark AE, et al (2005) GROMACS: fast, flexible, and free. J Comput Chem 26: 1701–1718 Available: http://www.ncbi.nlm.nih.gov/pubmed/16211538 Accessed 27 February 2013. 16211538 10.1002/jcc.20291

[39] Oostenbrink C, Villa A, Mark AE, Van Gunsteren WF (2004) A biomolecular force field based on the free enthalpy of hydration and solvation: the GROMOS force-field parameter sets 53A5 and 53A6. J Comput Chem 25: 1656–1676 Available: http://www.ncbi.nlm.nih.gov/pubmed/15264259 Accessed 4 November 2012. 15264259 10.1002/jcc.20090

[40] Oostenbrink C, Soares T a, van der Vegt NFA, Van Gunsteren WF (2005) Validation of the 53A6 GROMOS force field. Eur Biophys J 34: 273–284 Available: http://www.ncbi.nlm.nih.gov/pubmed/15803330. 15803330 10.1007/s00249-004-0448-6

[41] Bussi G, Donadio D, Parrinello M (2007) Canonical sampling through velocity-rescaling. J Chem Phys 126: 014101 Available: http://arxiv.org/abs/0803.4060 Accessed 4 December 2012. 17212484 10.1063/1.2408420

[42] Berendsen HJC, Postma JPM, Van Gunsteren WF, DiNola A, Haak JR (1984) Molecular dynamics with coupling to an external bath. J Chem Phys 81: 3684–3690 Available: http://link.aip.org/link/JCPSA6/v81/i8/p3684/s1 Accessed 4 December 2012.

[43] Berk Hess, Henk Bekker, Herman J. C Berendsen, Johannes G. E. M Fraaije (1997) LINCS: A linear constraint solver for molecular simulations. J Comput Chem 18: 1463–1472.

[44] Humphrey W, Dalke a, Schulten K (1996) VMD: visual molecular dynamics. J Mol Graph 14: 33–38, 27–28. Available: http://www.ncbi.nlm.nih.gov/pubmed/8744570. 10.1016/0263-7855(96)00018-5 8744570

[45] Eisenhaber F, Lijnzaad P, Argos P, Sander C, Scharf M (1995) The double cubic lattice method: Efficient approaches to numerical integration of surface area and volume and to dot surface contouring of molecular assemblies. J Comput Chem 16: 273–284 Available: http://doi.wiley.com/10.1002/jcc.540160303 Accessed 10 March 2014.

[46] Eisenberg D, McLachlan AD (1986) Solvation energy in protein folding and binding. Nature 319: 199–203 Available: http://www.nature.com/nature/journal/v319/n6050/abs/319199a0.html Accessed 11 December 2013. 3945310 10.1038/319199a0

[47] Kabach W, Sander C, Kabsch W (1983) Dictionary of protein secondary structure: pattern recognition of hydrogen-bonded and geometrical features. Biopolymers 22: 2577–2637 Available: http://www.ncbi.nlm.nih.gov/pubmed/6667333 Accessed 1 March 2013. 6667333 10.1002/bip.360221211

[48] Joosten RRP, te Beek T a H, Krieger E, Hekkelman ML, Hooft RWW, et al (2011) A series of PDB related databases for everyday needs. Nucleic Acids Res 39: D411–D419 Available: http://nar.oxfordjournals.org/content/39/suppl_1/D411.short Accessed 4 December 2012. 21071423 10.1093/nar/gkq1105 PMC3013697

[49] Frishman D, Argos P (1995) Knowledge-based protein secondary structure assignment. Struct Funct Bioinforma 23: 566–579. 10.1002/prot.340230412 8749853

[50] Heinig M, Frishman D (2004) STRIDE: a web server for secondary structure assignment from known atomic coordinates of proteins. Nucleic Acids Res 32: W500–W502 Available: http://nar.oxfordjournals.org/content/32/suppl_2/W500.short Accessed 4 December 2012. 15215436 10.1093/nar/gkh429 PMC441567

[51] Laskowski RA, MacArthur MW, Moss DS, Thornton JM (1993) PROCHECK: a program to check the stereochemical quality of protein structures. J Appl Crystallogr 26: 283–291 Available: http://scripts.iucr.org/cgi-bin/paper?S0021889892009944 Accessed 8 November 2013.

