# Supplementary material for: Investigation of the Josephin Domain Protein-Protein Interaction by Molecular Dynamics
Source: PLoS One. 2014 Sep 30;9(9):e108677. doi: 10.1371/journal.pone.0108677 (PMC4182536; doi:10.1371/journal.pone.0108677)
Supplement: File S1 — Figures, tables and additional information to support “MATERIALS AND METHODS” and “RESULTS” sections of this paper. (PDF) [file pone.0108677.s001.pdf]

SUPPORTING INFORMATION

S1. Molecular Dynamics Simulations

S1.1 JD^{Wat} simulations

MD simulations over three replicas of JD alone in water environment were carried out for 150 ns in the NVT ensemble. The C-alpha Root Mean Square Deviation of the whole protein is shown in Figure S1. Figure S2 highlights the contribution to the C-alpha RMSD given by JD globular core (hairpin and terminals excluded) and hairpin region (Val31-Leu62). Protein/Cα RMSD values ranging from 0.5 nm to 0.7 nm are detected for each simulated model (Figure S1), with a reasonable stability to the RMSD for the protein core (Figure S2a) in the last 50 ns. In all cases, the variation of the RMSD value for the globular core, is less than 0.1 nm, whereas the hairpin region (Val31-Leu62) is characterized by higher continuous RMSD fluctuations (Figure S2b).

The Ramachandran Plot of the JD provides structural validation of the obtained configuration, showing that more than 97% of the protein residues are in the most-favoured and additional-allowed regions (Figure S3).

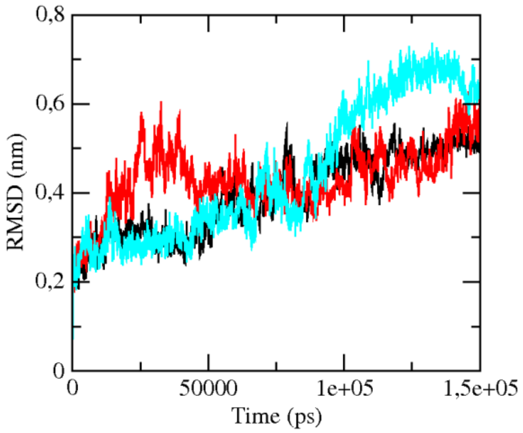

Figure S1. Protein/C-alpha Root Mean Square Deviation (RMSD) of the Josephin Domain alone in water environment (JD^{Wat} simulations, three replicas).

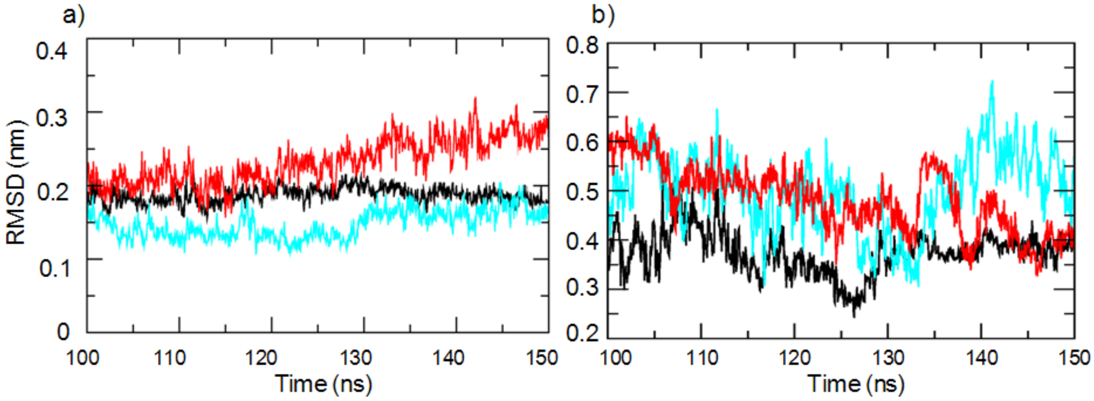

Figure S2. RMSD for JD^{Wat} simulations: a) protein core (hairpin and terminals excluded), b) hairpin domain.

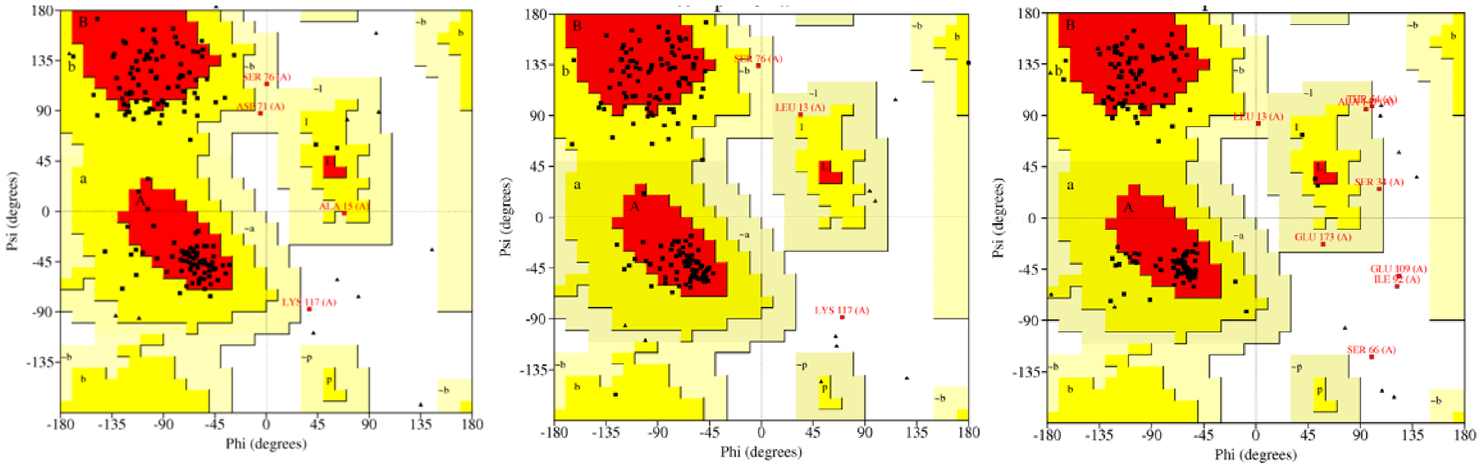

Figure S3. Ramachandran Plots of JDs (JD^{Wat} simulations).

S1.2 JD-JD simulations

Ten JD-JD molecular dynamics simulations, corresponding to ten different initial JD-JD orientations, were carried out for 150 ns in the NVT ensemble. In order to analyze the starting conformations for any possible bias, the contact probability percentage was calculated, using the 10 JD-JD initial configurations (Figure S4a and Figure S4b). The proteins were initially placed at 1 nm. For this reason, the contact probability (distance cutoff of 0.28 nm) of first plot (Figure S4a) is zero everywhere. Increasing the contact threshold (1nm), it was observed that the residues within the distance cutoff are equally distributed. The average intermolecular distance between a selected residue and the closest residue of the neighboring molecule is shown in Figure S4c, highlighting that all residues are placed, on average, at the same intermolecular distance (roughly 2nm).

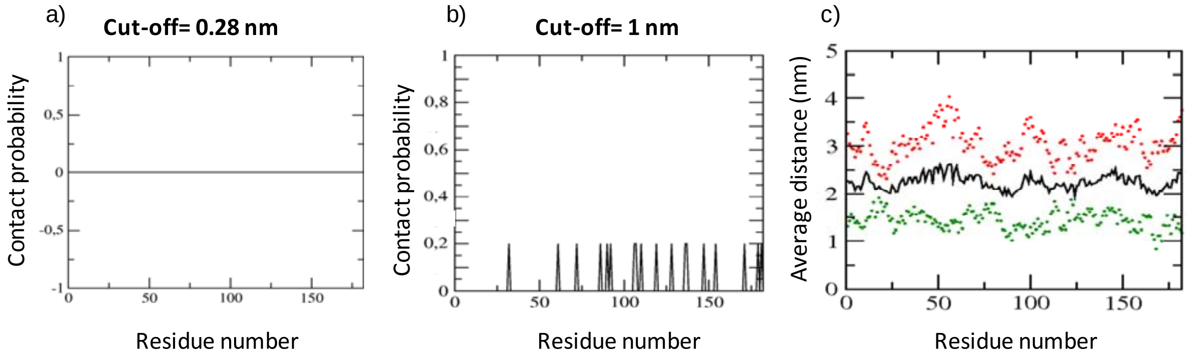

Figure S4. a) JD-JD residues contact probability with a distance cutoff of 0.28 nm. b) JD-JD residues contact probability with a distance cutoff of 1 nm. c) average (along the 10 configurations) inter-monomer residue distance from the interacting JD outer surface (black curve) . Green and red dotted curves should be interpreted as error bars of the black curve.

The stability of the JD-JD complex is indicated by the contact surface plots (Figure S5), with values ranging from 2.5 to 11 nm^2 in the last 50 ns of the simulations.

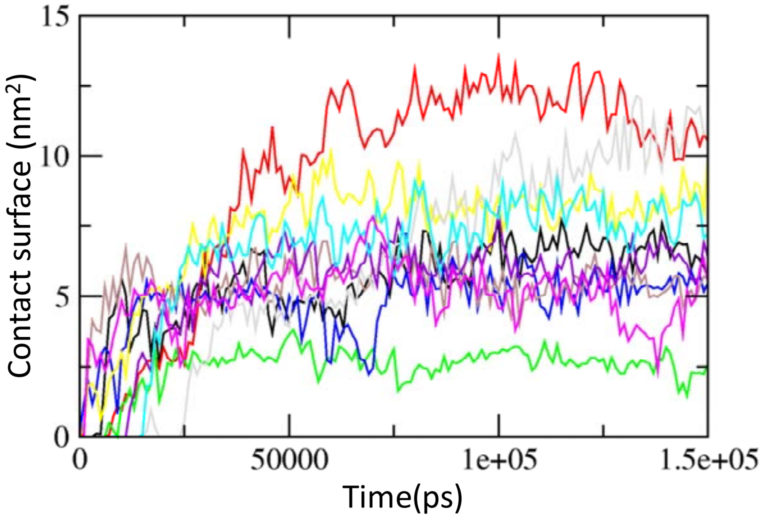

Figure S5. JD-JD contact surfaces throughout the MD simulations.

The MD simulations have allowed the identification of residues involved in the JD-JD contact surface: Arg101, Lys128, and Asp145. Also C-terminal (in particular Leu178/Arg182), seems to be involved in the JD-JD interaction interface with a contact probability around 40%. In order to provide more information about the Arg101, Lys128, Asp145 and Leu178/Arg182 binding interface, we have calculated the contact probability (Figure S6) for single residues. This has been calculated as the number of contact snapshots (e.g., distance between Arg101 on one monomer and all residues on the interfacing monomer through all JD simulations) divided by the total number of snapshots taken for the calculation (one snapshot every 50 ps between 100 and 150 ns for each JD-JD configuration). Figure 6S shows that Asp57 has been found to be the residue most frequently involved in the Arg101 binding interface (notice that Arg101 and Arg103 are very close to each other, showing contacts with almost identical residues on the interfacing monomer). Around 50% of JD-JD contact interfaces presenting Arg101 on one side show Asp57 on the contact interface of the facing monomer. The results shown in Figure S6 are summarized in Table S1, reporting the residues interacting with Arg101, Lys128, Asp145 and Leu178/Arg182. The interacting interface of Arg101 is mainly characterized by charged and polar residues (indicated in red and pink in the table). Therefore, JD-JD dimerization seems to be mainly driven by electrostatic interactions, as for example positively charged Arg101/Arg103 of one monomer and negative Asp57 of the second one.

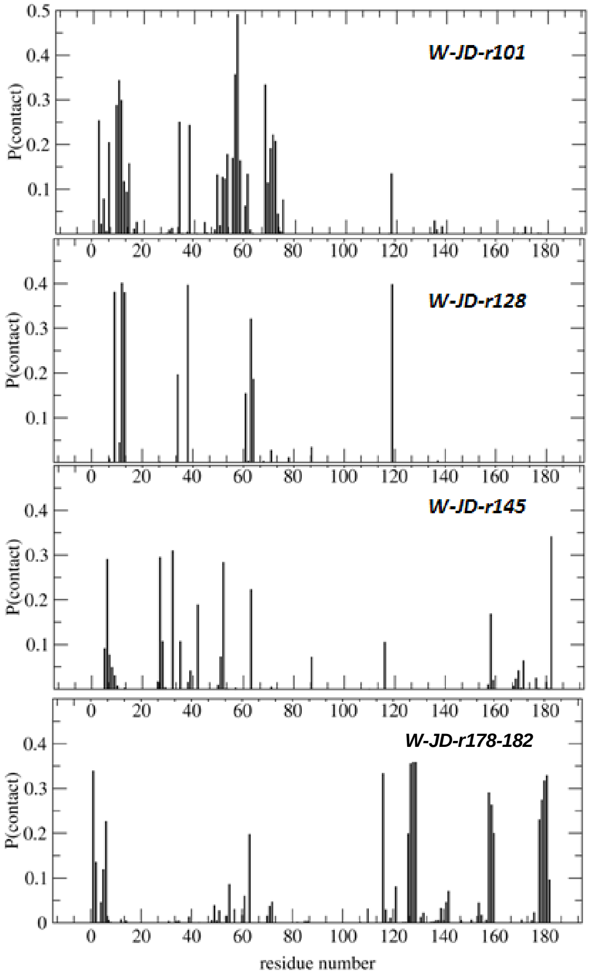

Figure S6. Contact probability calculated for all residues pertaining to a single JD with respect to residues Arg101,Lys128,Asp145, Leu178/Arg182 on the facing monomer for JDJD simulations.

Table S1. For all residues identified as mainly present in protein-protein contact surfaces amino acids on the interacting monomer are reported. From the left to the right, "*Residues in contact on the interacting monomer*" are ordered from the highest to the lowest contact probability.

Residue on JD		*Residues in contact on the interacting monomer*
Wild	*ARG-101*	*ASP-57, GLU-56, GLU-10, ASN-68, GLY-11, GLN-9, GLU-2, SER-34, HIS-38, ASP-71, SER-72, HIS-6*
Wild	*LYS-128*	*ARG-182, GLU-32, TYR-27, HIS-6, GLY-52, GLN-63*
Wild	*ASP-145*	*SER-12, HIS-119, HIS-38, GLN-9, LEU-13, GLN-63*
Wild	*LEU-178/ARG-182*	*GLN-129, LYS-128, GLY-127, MET-1, TYR-116, ILE-181, MET-180, GLU-158, GLN-179, GLY-159, LEU-178, HIS-6*

S1.3 JD$^{A101/103}$ simulations

Ten JD$^{A101/103}$ molecular dynamics simulations, corresponding to ten different initial JD-JD orientations, were carried out for 150 ns in the NVT ensemble. The JD$^{A101/103}$ simulations were performed starting from the same initial configuration of the wild type JD-JD system. For this reason, Figure S4 is representative for both JD-JD and

JD$^{A101/A103}$ simulations. The MD simulations have allowed the identification of the residues involved in the JD$^{A101/103}$ contact surface: Gln176-Arg182. In order to provide more information about the Gln176-Arg182 binding interface, we have calculated the corresponding contact probabilities (Figure S7). The residues Gln176, Gln179, Arg110, Tyr147, Ile181, Asp168, Glu90, Met1, Leu177, Arg182, Gln154 and Lys85 were identified as mainly involved in the Gln176-Arg182 binding interface (Table S2). It worth mentioning that 50% of interacting residues are from the C-terminus of the interfacing monomer. Hence, the mutated JD-JD interface is principally characterized by tail-tail interactions.

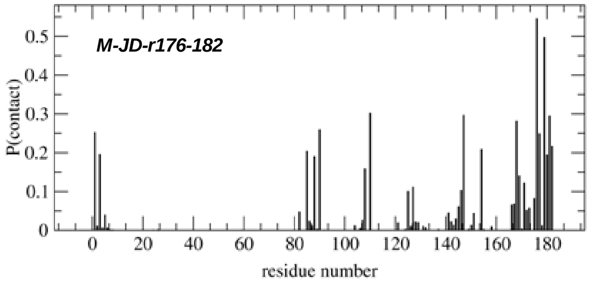

Figure S7. Contact probability calculated for all residues pertaining to a single JD with respect to Gln176/Arg182 on the facing monomer for JD$^{A101/103}$ simulations.

Table S2. For all residues identified as mainly present in protein-protein contact surfaces amino acids on the interacting monomer are reported. From the left to the right, "*Residues in contact on the interacting monomer*" are ordered from the highest to the lowest contact probability.

Residue on JD		*Residues in contact on the interacting monomer*
Mut	*GLN-176/ARG-182*	*GLN-176, GLN-179, ARG-110, TYR-147, ILE-181, ASP-168, GLU-90, MET-1, LEU-177, ARG-182, GLN-154, LYS-85*

S2. Fluctuations of Asp57-Leu62 (called α3) and relationship to the hairpin opening/closure dynamics.

Figure S8 shows α3 RMSF values, which are very different in a single JD structure than in an aggregated JD pair (wild or mutant). The reason for the different RMSF values between the single and the aggregated JD (mutant and wild type) might be due to the closure dynamics of the hairpin. In particular, the protein structural ensemble of the JD-JD and $JD^{A101/A103}$ includes JD configurations characterized by the hairpin in open-like, closed-like and intermediate state (Figure S8). It is worth noting that only the intermediate state is responsible for higher fluctuation values, with respect the open-like and the closed-like conformation. Hence, our results suggest α3 fluctuations to be directly related to hairpin opening/closure dynamics.

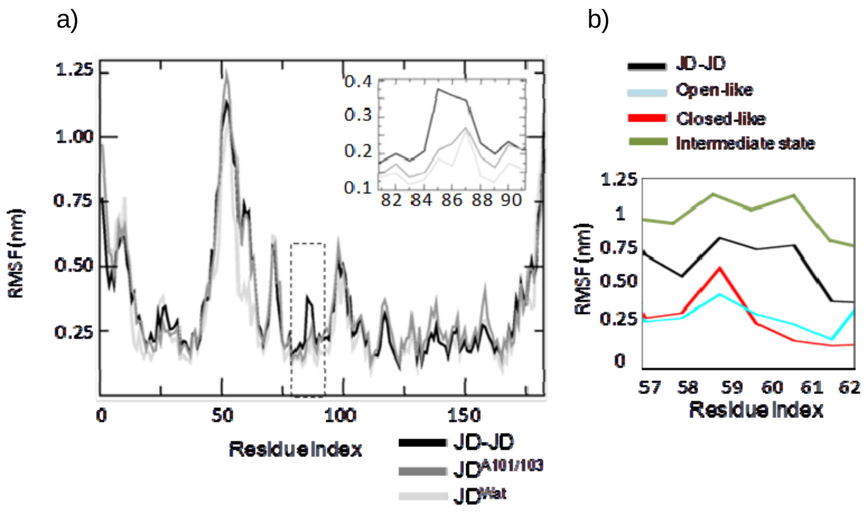

Figure S8. a) (Figure 5 of the manuscript) Single JD root-mean-square fluctuations (RMSF). Fluctuations of α4 are zoomed in the top-right panel where it is worth noticing that residues Leu84-Trp87 increase the RMSF as a consequence of JD dimerization (black curve). After Ala-mutation (gray curve) on Arg101-/Arg103 the RMSF value decreases to the value achieved by JD^{Wat} (light gray curve). b) A zoomed-in view of residues Asp57-Leu62. The RMSF has been calculated by dividing the snapshots through the open-like (cyan) hairpin JD structure, closed like (red) hairpin JD structure, intermediate (green) hairpin JD structure. In black is reported the average RMSF curve for JD-JD.
